# Worsening and newly diagnosed paraneoplastic syndromes following anti-PD-1 or anti-PD-L1 immunotherapies, a descriptive study

**DOI:** 10.1186/s40425-019-0821-8

**Published:** 2019-12-03

**Authors:** Guillaume Manson, Alexandre Thibault Jacques Maria, Florence Poizeau, François-Xavier Danlos, Marie Kostine, Solenn Brosseau, Sandrine Aspeslagh, Pauline Du Rusquec, Maxime Roger, Maud Pallix-Guyot, Marc Ruivard, Léa Dousset, Laurianne Grignou, Dimitri Psimaras, Johan Pluvy, Gilles Quéré, Franck Grados, Fanny Duval, Frederic Bourdain, Gwenola Maigne, Julie Perrin, Benoit Godbert, Beatris Irina Taifas, Alexandra Forestier, Anne-Laure Voisin, Patricia Martin-Romano, Capucine Baldini, Aurélien Marabelle, Christophe Massard, Jérôme Honnorat, Olivier Lambotte, Jean-Marie Michot

**Affiliations:** 1 Département d’Innovation Thérapeutique et d’Essais Précoces, Gustave Roussy, Université Paris-Saclay, F-94805 Villejuif, France; 2 Department of Hematology, University Hospital of Rennes, Rennes, France; 3 Department of Internal Medicine and Multiorgan Diseases, Referral Center for Auto-immune Diseases, Saint-Eloi Hospital Montpellier University, Montpellier, France; 4 Department of Dermatology, Rennes University Hospital, Rennes, France; 5 Rheumatology Department, Bordeaux University Hospital, Bordeaux, France; 6 AP-HP, Hôpital Bichat-Claude Bernard, Centre Investigation Clinique 1425, Thoracic Oncology Department, University Paris-Diderot, Paris, France; 7 Department of Medical Oncology, Universitair Ziekenhuis Brussel, Brussels, Belgium; 8 Medical Oncology Department, Institut de Cancérologie de l’Ouest, Centre René Gauducheau, Saint-Herblain, France; 9 Department of Pulmonology and Thoracic Oncology, Rouen University Hospital, Rouen, France; 10 Neurology Department, Orléans Hospital, Orléans, France; 11 Internal Medicine Department, Clermont-Ferrand University Hospital, Clermont-Ferrand, France; 12 Dermatology Department, Bordeaux University Hospital, Bordeaux, France; 13 Neurology Department, Brest University Hospital, Brest, France; 14 AP-HP, Groupe Hospitalier Pitié-Salpêtrière, Service de Neurologie 2-Mazarin et Université Pierre et Marie Curie-Paris 6, Centre de Compétence des Syndromes Neurologiques Paranéoplasiques et Encéphalites Auto-immunes, Paris, France; 15 Oncology Departement, Brest Hôpital Morvan Centre Hospitalier Régional Universitaire, Brest, France; 16 Amiens University Hospital, Rheumatology Department, University of Picardie - Jules Verne, Amiens, France; 17 Neurology Department, Bordeaux University Hospital, Bordeaux, France; 18 Departement de Neurologie, Centre Hospitalier de la Côte Basque, Bayonne, France; 19 Department of Internal Medicine, Caen University Hospital, Caen, France; 20 Pneumology Department, Metz Robert Schuman Hospital, Metz, France; 21 Hôpital d’Instruction des Armées Percy, Service de Neurologie, Clamart, France; 22 Oncology Department, Centre Oscar Lambret, Lille, France; 23 Gustave Roussy, Université Paris-Saclay, Unité fonctionnelle de Pharmacovigilance, F-94805 Villejuif, France; 24 Hospices Civils de Lyon, French Reference Center on Paraneoplastic Neurological Syndromes and Autoimmune Encephalitis, SynatAc Team, Institut NeuroMyoGène. INSERM U1217/CNRS UMR 5310, Université Claude Bernard Lyon 1, Université de Lyon, Lyon, France; 25 AP-HP, Hôpital Bicêtre, Service de Médecine Interne et Immunologie Clinique, Le Kremlin-Bicêtre, France; 26 Université Paris Sud, Centre de Recherche en Immunologie des Infections Virales et des Maladies Auto-Immunes, INSERM U1184, Le Kremlin-Bicêtre, France; 27 Division d’Immunovirologie, Commissariat à l’Energie Atomique et aux Energies Alternatives, Fontenay-aux- Roses, France

## Abstract

**Background:**

Paraneoplastic syndromes (PNS) are autoimmune disorders specifically associated with cancer. There are few data on anti-PD-1 or anti-PD-L1 immunotherapy in patients with a PNS. Our objective was to describe the outcome for patients with a pre-existing or newly diagnosed PNS following the initiation of anti-PD-1 or anti-PD-L1 immunotherapy.

**Methods:**

We included all adult patients (aged ≥18) treated with anti-PD-1 or anti-PD-L1 immunotherapy for a solid tumor, diagnosed with a PNS, and registered in French pharmacovigilance databases. Patients were allocated to cohorts 1 and 2 if the PNS had been diagnosed before vs. after the initiation of immunotherapy, respectively.

**Findings:**

Of the 1304 adult patients screened between June 27th, 2014, and January 2nd, 2019, 32 (2.45%) had a PNS and were allocated to either cohort 1 (*n* = 16) or cohort 2 (*n* = 16). The median (range) age was 64 (45–88). The tumor types were non-small-cell lung cancer (*n* = 15, 47%), melanoma (*n* = 6, 19%), renal carcinoma (*n* = 3, 9%), and other malignancies (*n* = 8, 25%). Eleven (34%) patients presented with a neurologic PNS, nine (28%) had a rheumatologic PNS, eight (25%) had a connective tissue PNS, and four (13%) had other types of PNS. The highest severity grade for the PNS was 1–2 in 10 patients (31%) and ≥ 3 in 22 patients (69%). Four patients (13%) died as a result of the progression of a neurologic PNS (encephalitis in three cases, and Lambert-Eaton syndrome in one case). Following the initiation of immunotherapy, the PNS symptoms worsened in eight (50%) of the 16 patients in cohort 1.

**Interpretation:**

Our results show that PNSs tend to be worsened or revealed by anti-PD-1 or anti-PD-L1 immunotherapy. Cases of paraneoplastic encephalitis are of notable concern, in view of their severity. When initiating immunotherapy, physicians should carefully monitor patients with a pre-existing PNS.

## Introduction

Over the last 5 years, anti-programmed cell death protein (PD)-1 or anti-programmed death-ligand (L)-1 immunotherapy has proven to be highly effective in the treatment of various types of cancer. By releasing the immune brake on antitumor activity, immunotherapy can also trigger immune-related adverse events (irAEs) [1]. Checkpoint blockade by an anti-PD-1 or anti-PD-L1 will induce an irAE in around 40% of patients, and 8% of patients will experience a severe (grade 3 or 4) irAE [2, 3]. There are two main types of irAE: the first, most frequently observed type is an immune-mediated inflammation that in principle can affect any organ (the thyroid, lungs, skin, digestive tract, etc.) [1], and the second corresponds to a flare-up of a pre-existing autoimmune disorder [4–6].

Paraneoplastic syndromes (PNSs) are rare autoimmune disorders associated with cancer [7, 8]. The complex pathogenesis of these syndromes is mediated by either soluble factors (such as hormones or cytokines secreted by the tumor) or cellular immune mechanisms that target tumor cells displaying cross-reactivity with self antigens [8–10]. To date, little is known about the use of immune checkpoint inhibitor in patients with a PNS, although some recent data suggest that these syndromes are complications of immunotherapy [10–13]. It is noteworthy that PNSs are generally associated with specific tumor types that are not routinely treated with immune checkpoint inhibitors; these include small-cell lung cancers [14], gynecological cancers [15], and thymic carcinomas [16]. A recent review of neurologic PNSs that occurred following treatment with immune checkpoint inhibitors emphasized the clinical seriousness of these syndromes and the need for further investigation in the context of immunotherapy [11]. Here, we performed a French, nationwide, observational, multicenter study of patients with PNS having undergone anti-PD-1 or anti-PD-L1 immunotherapy.

## Methods

### Study design and participants

**Fig. 1:**
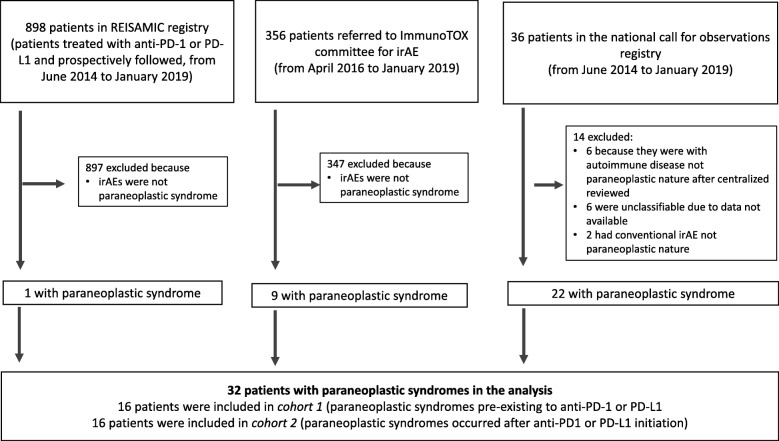
Study flow chart. irAE: immune-related adverse event

This was a French, nationwide, observational study of adult patients (aged 18 or over) presenting with a PNS before or after the initiation of anti-PD-1 or anti-PD-L1 immunotherapy for a solid tumor. The patients were recruited via three registries: (i) the French REISAMIC pharmacovigilance registry (*Registre des Effets Indésirables Sévères des Anticorps Monoclonaux Immunomodulateurs en Cancérologie* [3]) between June 27th, 2014, and January 2nd, 2019, (ii) the ImmunoTOX toxicity committee at the Gustave Roussy cancer center (Villejuif, France) [17] between April 6th, 2016, and January 2nd, 2019, and (iii) a French nationwide call for observations via the *Société Nationale Française de Médecine Interne* (SNFMI) and the *Club Rhumatisme et Inflammation* (CRI) learned societies in January 2019. In the latter call, we asked for observations of patients with a pre-existing or newly diagnosed PNS following anti-PD1 or anti-PD-L1 immunotherapy between June 27th, 2014, and January 2nd, 2019 (Fig. 1).

Patients with PNS were then allocated to one of two observational cohorts. Cohort 1 comprised patients diagnosed with a PNS prior to the initiation of anti-PD-1 or anti-PD-L1 immunotherapy, whereas cohort 2 comprised patients with a PNS diagnosed after the initiation of anti-PD-1 or anti-PD-L1 immunotherapy. The study’s primary objective was to describe the outcome of the PNSs reported in the surveyed databases. The secondary objectives were to report the time interval between the initiation of immunotherapy and the exacerbation or appearance of the PNS, the frequency with which pre-existing PNSs were exacerbated, and the treatment of the PNSs.

### Study procedures

The REISAMIC registry is an academic-led pharmacovigilance database that was set up at Gustave Roussy on June 27th, 2014. The goal is to collate and investigate all grade ≥ 2 irAEs (according to the Common Terminology Criteria for Adverse Events (CTCAE), version 4.03) related to anti-PD-1 or anti-PD-L1 immunotherapy, and thus improve the management of these events in routine clinical practice [3]. The registry includes all patients aged 18 or over having received anti-PD-1 or anti-PD-L1 agents for a solid tumor at Gustave Roussy, regardless of their estimated survival time. The ImmunoTOX committee is an academic board of oncologists, internists and organ specialists based at Gustave Roussy, and was set up on April 6th, 2016 [17]. The committee’s goal is to help oncologists manage irAEs in clinical practice.

The severity of each PNS was assessed according to the CTCAE v4.03 guidelines. The CTCAE grade severity on a scale of 1 to 5, and gives a clinical description of severity for each adverse event. A panel of 26 different types of PNS was predefined, according to Henry’s classification [8] (Additional file 1: Table S1). To enter the study, patients had to have at least one type of predefined PNS. In all cases, the treating physician had to have filled out a comprehensive pharmacovigilance report. All PNSs recorded were reviewed centrally and were confirmed by a committee of physicians with expertise in the management of PNSs and autoimmune disorders (OL, JH, Al.M, JMM, and GM). This expert committee reviewed the following data: the characteristics of the immunotherapy regimen, the clinical characteristics of the PNS, the results of serologic assays for autoimmune factors (when performed), the medications administered to treat the PNS, the PNS’s highest grade of severity, and the clinical outcome.

### Outcome

The follow-up period was defined as the time interval between the initiation of anti-PD-1 or anti-PD-L1 immunotherapy and last follow-up or all-cause death. Antitumor responses following anti-PD-1 or anti-PD-L1 immunotherapy were recorded and assessed by the investigators according to the Response Evaluation Criteria in Solid Tumors (version 1.1), as modified for use in clinical trials of immune checkpoint inhibitors [18]. The antitumor response was first recorded when the PNS worsened or was first diagnosed. We also noted the best antitumor response recorded during the patient’s regular CT assessments (scheduled every two or three months, depending on the immunotherapy used).

### Statistical analysis

Data were quoted as the median (range). Adverse events and PNSs were stratified by severity (grades 1–2, 3–4, and 5). All patients gave their verbal, informed consent to participation in the study. The study was approved by the institutional review board at Gustave Roussy, and the REISAMIC registry was registered with the French Data Protection Commission (*Commission Nationale de l’Informatique et des Libertés*, Paris, France; reference number 2098694v0).

## Results

### Patient recruitment

Of the 1290 patients screened in the pharmacovigilance databases (898 patients from the REISAMIC registry, 356 from the ImmunoTOX committee, and 36 from the nationwide call for observations), 32 (2.45%) patients were selected for analysis after the central review and were allocated to cohort 1 (*n* = 16 patients) or cohort 2 (*n* = 16 patients). Between April 1st, 2016, and January 2nd, 2019, the ImmunoTOX committee registered 356 referrals for advice, of which nine (2.53%) concerned a patient with PNS. The national call for observations via the SNFMI and the CRI learned societies generated 36 reports of patients with PNS. After the central review, 14 patients were excluded from the analysis because (i) they were considered to have a nonparaneoplastic autoimmune disease or a nonautommune irAE in the central review, or (ii) a lack of data prevented a firm diagnosis (Fig. 1).

### Clinic characteristics of the study population

The clinical characteristics of the 32 patients (21 males (66%)) are summarized in Table 1. The median (range) age was 64 (45–88). The tumor types were non-small-cell lung cancer (*n* = 15, 47%), melanoma (*n* = 6, 19%), renal carcinoma (*n* = 3, 9%), and other malignancies (*n* = 8, 25%). Prior to immunotherapy, patients had received a median (range) of one course of systemic cancer treatment (0–5). Twenty-eight patients (88%) had received anti-PD-1 monotherapy, three patients (9%) had received anti-PD-L1 monotherapy, and one patient (3%) had received a combination of an anti-PD-1 agent and an anti-CTLA4 agent. The median (range) length of follow-up after the initiation of immunotherapy was 9.6 months (0.9–17.7). Overall, 47% of the patients achieved an objective antitumor response at some point during the follow-up period, and 9% of the patients achieved a complete response.

**Table 1:** Characteristics of patients with paraneoplastic syndromes selected for analysis after central review. Patients were included in two observational cohorts, defined as follows: cohort 1 comprised patients with a PNS diagnosed before the initiation of immunotherapy, and cohort 2 comprised patients with a PNS diagnosed after the initiation of immunotherapy

Patient characteristics	Cohort 1 (pre-existing PNS) *n* = 16 patients	Cohort 2 (newly diagnosed PNS) *n* = 16 patients	Total *n* = 32 patients (%)
Age, in years, median (range)	64 (48–86)	68 (45–88)	64 (45–88)
Sex ratio (male/female)	4.3	0.5	1.8
Tumor type, n patients (%)			
- NSCLC	10	5	15 (47)
- Melanoma	1	5	6 (19)
- Renal carcinoma	1	2	3 (9)
- Merkel carcinoma	1	1	2 (6)
- Neuroendocrine carcinoma	1	0	1 (3)
- Pulmonary sarcomatoid carcinoma	1	0	1 (3)
- HNSCC	1	0	1 (3)
- Esthesioneuroblastoma	0	1	1 (3)
- Mesothelioma	0	1	1 (3)
- Breast cancer	0	1	1 (3)
Number of prior courses of systemic cancer treatment, median (range)	1.0 (0–2)	0.5 (0–5)	1.0 (0–5)
Immunotherapy received, n (%)			
- Anti-PD-1	**14 (88)**	**14 (88)**	**28 (88)**
- Nivolumab	9	9	18
- Pembrolizumab	5	5	10
- Anti-PDL-1	**2 (12)**	**1 (6)**	**3 (9)**
- Avelumab	1	1	2
- Durvalumab	1	0	1
- Combination immunotherapy			
- Nivolumab + ipilimumab	**0**	**1 (6)**	**1 (3)**
Best overall antitumor response during immunotherapy, n (%)^a^			
- Objective response	**9 (56)**	**6 (38)**	**15 (47)**
- CR	2 (12)	1 (6)	3 (9)
- PR	7 (44)	5 (31)	12 (38)
- SD	**3 (19)**	**8 (50)**	11 (34)
- PD	**4 (25)**	**2 (13)**	6 (19)
Immune-related adverse events other than PNSs (all severity grades), n (%)^b^	**4 (25)**	**6 (38)**	**10 (31)**
- Dysthyroidism	1	1	2 (6)
- Vitiligo	0	2	2 (6)
- Hepatitis	0	1	1 (3)
- Oligo-arthritis	1	0	1 (3)
- Myocarditis	1	0	1 (3)
- Rash	1	0	1 (3)
- Pneumonitis	0	1	1 (3)
- Fever	0	1	1 (3)
Length of follow-up after immunotherapy initiation, median (range), months	7.9 (0.9;17.7)	10.5 (4;17.4)	9.6 (0.9;17.7)

*CR* Complete response, *HNSCC* Head and neck squamous cell carcinoma, *NSCLC* Non-small-cell lung carcinoma, *PD* Progressive disease, *PNS* Paraneoplastic syndrome, *PR* Partial response, *SD* Stable disease

^a^According to the iRECIST criteria. In patients allocated to cohort 1 who did not experienced worsening of PNS, the best overall response is shown

^b^All-grade severity, according to the CTCAE v4.03

### Characteristics and severity of the PNSs

The characteristics of the PNSs and the patients’ clinical signs and symptoms are summarized in Table 2. Overall, 11 of the 32 patients (34%) had a neurologic PNS, nine (28%) had a rheumatologic PNS, eight (25%) had a connective tissue PNS, and four (13%) had another type of PNS (detailed in Table 2**)**. The highest CTCAE severity grade was grade ≥ 3 in 22 (69%) patients: 18 patients had a grade 3–4 event, and four of the 32 patients (13%) had a grade 5 event (i.e. resulting in death). Overall, nine (28%) patients died: four deaths were related to the PNS, four patients died from tumor progression, and one patient (included in cohort 1 because of pre-existing dermatomyositis) died after suffering an ischemic stroke with hemorrhagic transformation not related to the immunotherapy or tumor progression.

**Table 2:** Main characteristics of PNSs experienced by patients selected for analysis after central review

Results	Patients with pre-existing PNS (cohort 1), *n* = 16	Patients with newly diagnosed PNS (cohort 2), *n* = 16	Total patients *n* = 32
Time from diagnosis of cancer to diagnosis of the PNS, median (range), months	0.3 (−62.8;406.2)^a^	18.6 (3.9;281.5)	11.9 (−62.8;406.2)
Neurologic PNS, n patients (%)	**4 (25)**	**7 (44)**	11 (34)
- Encephalitis	1	5	6
- Neuropathy	2	1	3
- Lambert-Eaton syndrome	1	1	2
Rheumatologic PNS, n patients (%)	3 (19)	6 (38)	9 (28)
- Hypertrophic osteoarthropathy	2	4	6
- RS3PE	0	2	2
- Rhizomelic pseudopolyarthritis	1	0	1
Connective tissue PNS, n patients (%)	6 (37)	2 (12)	8 (25)
- Dermatomyositis	4	1	5
- Systemic sclerosis	1	1	2
- Myositis (anti-PL7 antisynthetase syndrome)	1	0	1
Other PNSs, n patients (%)	3 (19)	1 (6)	4 (13)
- Membranous nephropathy	1	0	1
- IgA vasculitis or Henoch-Schönlein purpura	1	0	1
- Other, thrombotic microangiopathy	1	0	1
- Other, Cushing’s disease	0	1	1
Highest CTCAE grade for PNS severity, n of patients (%)			
- Grade 1–2	5 (31)	5 (31)	10 (31)
- Grade 3–4	11 (69)	7 (43)	18 (56)
- Grade 5	0	4 (25)	4 (13)
CTCAE grade for PNS severity at last follow-up, n of patients (%)			
- Grade 0–1	7 (44)	5 (31)	12 (38)
- Grade ≥ 2	9 (56)	11 (69)	20 (62)
Causes of death, n of patients (%)	**3 (19)**	**6 (38)**	**9 (28)**
- PNS	0	4	4 (13)
- tumor progression	2	2	4 (13)
- comorbidity	1	0	1 (3)

*CTCAE* Common Terminology Criteria for Adverse Events, *PNS* paraneoplastic syndrome, *RS3PE* remitting seronegative symmetrical synovitis with pitting edema

^**a**^Some patients presented with a PNS before the cancer diagnosis, which explains the negative lower boundary. Some patients presented with PNS at cancer relapse, which explains why the time between cancer diagnosis and PNS exacerbation was sometimes greater than 60 months

The outcomes for the 24 patients with an exacerbated pre-existing PNS (8 of the 16 patients in cohort 1) or with a newly diagnosed PNS (all 16 patients in cohort 2) are shown in Table 3. The median (range) time between the start of immunotherapy and the PNS exacerbation or new diagnosis was 1.4 months (0.5–6.4). The exacerbation or new diagnosis prompted the temporary withdrawal of immunotherapy in 6 patients (25%) and the permanent withdrawal of immunotherapy in 14 patients (58%). The remaining 4 patients (17%) continued their anti-PD1 or anti-PD-L1 immunotherapy as planned.

**Table 3:** Characteristics of patients with worsening pre-existing PNS after immunotherapy (cohort 1) or newly diagnosed PNS following immunotherapy (cohort 2)

Outcome of patients with worsening or newly diagnosed PNS	Cohort 1 (pre-existing PNS) *n* = 8 patients with PNS worsening	Cohort 2 (newly diagnosed PNS) *n* = 16 patients	Total *n* = 24 patients
Time from initiation of immunotherapy to PNS worsening (cohort 1) or new diagnosis of a PNS (cohort 2), median (range), in months	0.9 (0.5–2.8)	1.6 (0.5–6.4)	1.4 (0.5–6.4)
Antitumor response at time of worsening or newly diagnosed PNS, n (%)			
- CR	2 (25)	1 (6)	3 (13)
- PR	2 (25)	5 (31)	7 (30)
- SD	2 (25)	10 (63)	12 (50)
- PD	2 (25)	0	2 (8)
Best overall antitumor response			
- CR	2 (25)	1 (6)	3 (13)
- PR	3 (38)	5 (31)	8 (33)
- SD	1 (13)	8 (50)	9 (38)
- PD	2 (25)	2 (13)	4 (17)
Impact of PNS on immunotherapy, n (%)			
- temporary discontinuation	2 (25)	4 (25)	6 (25)
- permanent discontinuation	5 (62)	9 (56)	14 (58)
- no discontinuation	1 (13)	3 (19)	4 (17)

*CR* Complete response, *CTCAE* Common Terminology Criteria for Adverse Events, *PD* Progressive disease, *PNS* Paraneoplastic syndrome, *PR* Partial response, *SD* Stable disease

### Patients diagnosed with a PNS before the initiation of anti-PD-1 or anti-PD-L1 immunotherapy (cohort 1)

**Fig. 2:**
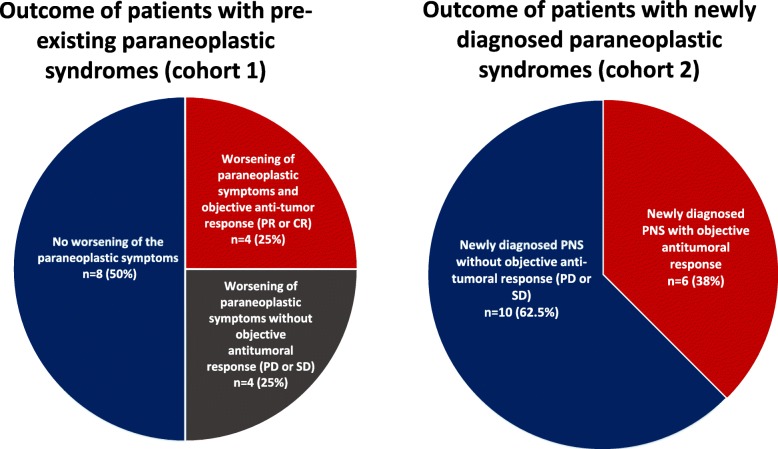
Outcomes (PNS symptoms and tumor responses) for patients diagnosed with a PNS before (cohort 1, left panel) or after (cohort 2, right panel) the initiation of immunotherapy. CR: Complete response. PR: Partial response. PNS: Paraneoplastic syndrome. PD: Progressive disease. SD: Stable disease

Sixteen patients had a pre-existing PNS, and so were included in cohort 1. Connective tissue PNSs were most frequent (*n* = 6 patients, 37%) (Table 2). In eight patients (50%), the PNS worsened after the initiation of anti-PD-1 or anti-PD-L1 immunotherapy. The median (range) time interval between anti-PD1 or anti-PD-L1 initiation and exacerbation of the PNS was 0.9 months (0.5–2.8) (Table 3). Four patients (25%) were found to have an objective antitumor response at the time of the exacerbation (Table 3 and Fig. 2). In cohort 1, exacerbation of the PNS during immunotherapy was not correlated with the concomitant tumor response (Additional file 2: Table S2).

### Patients diagnosed with a PNS after the initiation of anti-PD-1 or anti-PD-L1 immunotherapy (cohort 2)

**Fig. 3:**
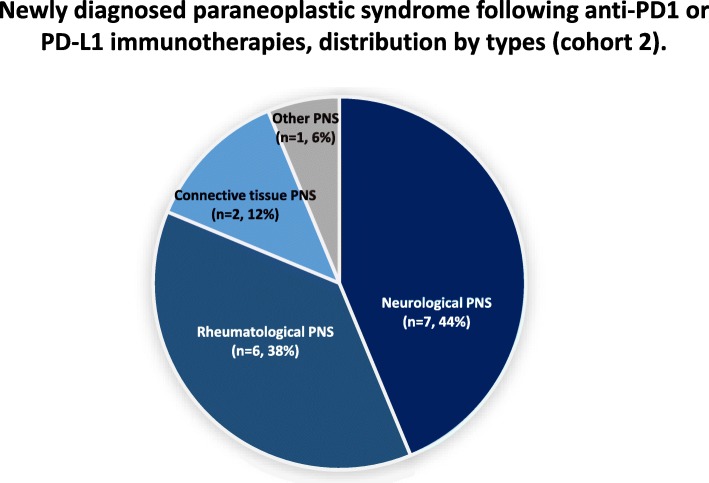
Types of PNS in patients diagnosed with the syndrome after the initiation of anti-PD-1 or anti-PD-L1 immunotherapy (cohort 2). PNS: paraneoplastic syndrome

Sixteen patients had a newly diagnosed PNS, and so were included in cohort 2. The most frequent PNS categories were neurologic conditions (*n* = 7, 44%) and rheumatologic conditions (*n* = 6, 38%) (Table 2 and Fig. 3). The median (range) time between immunotherapy initiation and the new diagnosis of a PNS was 1.6 months (0.5–6.4) (Table 3). Six (38%) patients had an objective tumor response at the time when the PNS appeared (Fig. 2).

### Characteristics of the PNSs (cohorts 1 and 2 together), by clinical type

The three most frequent clinical types of PNS were hypertrophic osteoarthropathy, encephalitis, and dermatomyositis (Table 4).

- **Hypertrophic osteoarthropathy**. Six patients had hypertrophic osteoarthropathy (two in cohort 1 and four in cohort 2). All six patients had received anti-PD1 monotherapy. The PNS was not severe, in most cases: four patients had a highest CTCAE grade ≤ 2, none of the patients permanently discontinued immunotherapy because of the PNS, and none died as a result of the PNS. All six patients were treated with steroids or nonsteroidal anti-inflammatory drugs, and the subsequent outcome was usually good (Table 4).
- **Encephalitis**. Six patients were recorded with paraneoplastic encephalitis (one in cohort 1 and five in cohort 2). Five patients had received anti-PD1 monotherapy, and the remaining patient had received a combination of anti-PD1 and anti-CTLA4 immunotherapy. Four of the six patients were positive for anti-Ma2 autoantibodies; in these cases, the tumors were variously non-small-cell lung carcinoma (*n* = 2), renal carcinoma (*n* = 1), and mesothelioma (*n* = 1). All six patients had severe neurologic symptoms, with a highest CTCAE grade ≥ 3. Three patients had a grade 5 event and died as a result of the paraneoplastic encephalitis. The PNS had prompted the permanent discontinuation of anti-PD-1 immunotherapy in all six patients. Steroids alone did not effectively control the encephalitis, and five of the six patients received additional immunomodulatory treatments (including plasma exchange, polyvalent immunoglobulins, cyclophosphamide, and rituximab; Table 4).
- **Dermatomyositis.** Five patients with dermatomyositis were assessed (four in cohort 1 and one in cohort 2). The dermatomyositis was exacerbated after immunotherapy in three of the four patients in cohort 1. Only one of the five patients was positive for anti-TIF1 antibodies. All five patients had a highest CTCAE grade ≥ 3, and none of the patients died as a result of the PNS. The dermatomyositis appeared or worsened within one month of initiating immunotherapy. Steroids were partly effective but additional immunosuppressants or immunomodulators (including oral methotrexate, polyvalent immunoglobulins, and plasma exchange) were administered to four of the five patients (Table 4).

**Table 4:** Characteristics and outcomes of patients with PNS, by types

Patient characteristics	Paraneoplastic hypertrophic osteoarthropathy (*n* = 6 pts)	Paraneoplastic encephalitis (*n* = 6 pts)	Paraneoplastic dermatomyositis (*n* = 5 pts)
Patients with a pre-existing PNS	2	1	4
Patients with a newly diagnosed PNS	4	5	1
Clinical type	• Hypertrophic osteoarthropathy (*n* = 6 pts)	• Anti-Ma2 autoantibody encephalitis (*n* = 4 pts) • Anti-neuron antibody encephalitis (*n* = 1 pt) • Cortical myoclonus encephalitis (*n* = 1 pt)	• Seronegative dermatomyositis (*n* = 4 pts) • Anti-TIF1-associated dermatomyositis (*n* = 1 pt)
Cancer type			
- NSCLC	4	3	2
- Pulmonary sarcomatoid carcinoma	1	0	0
- Renal carcinoma	1	1	0
- Mesothelioma	0	1	0
- Melanoma	0	1	2
- Neuroendocrine carcinoma	0	0	1
Bone metastasis	1	0	1
General outcome for the PNS following anti-PD1 or PD-L1 immunotherapy			
- No worsening	1	0	1
- Worsening	1	1	3
- Newly diagnosis of a PNS	4	5	1
Highest CTCAE grade of PNS severity			
- Grade 1–2	4	0	0
- Grade ≥ 3	2	6	5
Time interval between initiation of immunotherapy to worsening or new diagnosis of the PNS, median (range), months	1.4 (0.5–5)	2.6 (0.5–5.5)	0.7 (0.5–0.9)
Antitumor response at the time of worsening or newly diagnosis of a PNS			
- CR	0	0	1
- PR	0	4	1
- SD	5	2	0
- PD	1	0	0
- Not evaluated	0	0	2
Impact of paraneoplastic syndrome on immunotherapy, n			
- temporary discontinuation	3	0	1
- permanent discontinuation	0	6	3
- no discontinuation	3	0	1
Paraneoplastic syndrome treatment			
First-line treatment	All 5 patients received first-line treatment: - Steroids (*n* = 3): complete resolution in one patient, partial resolution in two patients - NSAIDs (*n* = 2): complete resolution in one patient, partial resolution in one patient	All 6 patients received first-line treatment: - Steroids (*n* = 5): partial resolution in four patients, no resolution in one patient. - Steroids plus immunoglobulins (*n* = 1): partial resolution in one patient three patients	All 4 patients received first-line treatment: - Steroids (*n* = 2): partial resolution in two patients - Steroids plus immunoglobulins (*n* = 2): partial resolution in one patient, no resolution in one patient.
Second-line treatments (if required)	Second-line treatment was required in one patient: - Methotrexate: partial resolution in one patient.	Second-line treatment was required in five patients: - Immunoglobulins (*n* = 2) partial response in two patients - Cyclophosphamide (*n* = 1): partial resolution in one patient - Rituximab (*n* = 1): partial resolution in one patient - Plasma exchange (*n* = 1): partial resolution in one patient	Second-line treatment was required in four patients: - Methotrexate (*n* = 3): partial resolution in three patients - Plasma exchange plus methotrexate (*n* = 1): partial resolution in one patient.
Persistent of PNS symptoms with a CTCAE grade > 1 at last follow-up, n (%)	1 (20)	5 (83)	3 (75)
PNS related death, n (%)	0	3 (50)	0

*CR* Complete response, *CTCAE* Common Terminology Criteria for Adverse Events, *NA* Not available, *NSAID* Non-steroidal anti-inflammatory drug, *NSCLC* Non-small-cell lung carcinoma, *PD* Progressive disease, *PNS* Paraneoplastic syndrome, *PR* Partial response, *Pt* patient, *SD* Stable disease

## Discussion

To the best of our knowledge, the present study is the first to have described the tolerability of immunotherapy in patients with a pre-existing or newly diagnosed PNS. We studied patients with neurologic (34%), rheumatologic (28%) and connective tissue PNSs (25%). Half of patients with a pre-existing PNS experienced a worsening of the corresponding symptoms after the initiation of anti-PD-1 or anti-PD-L1 immunotherapy. Our observations also highlighted the seriousness of PNSs (especially neurologic PNSs), since four of the 32 patients (13%) died (paraneoplastic encephalitis: *n* = 3; Lambert Eaton syndrome: *n* = 1).

For the study population as a whole (i.e. cohorts 1 and 2), the overall response rate of 47% was relatively high. Although our study’s descriptive, retrospective design prevents us from drawing robust conclusions about responses rates, this high response rate emphasizes that the exacerbation or appearance of a PNS can be associated with an effective tumor response soon after the initiation of immunotherapy.

Patients with a PNS accounted for 2.53% of all the requests addressed to the ImmunoTOX committee; hence, these syndromes are rare but are likely be encountered in routine clinical practice. The relatively low prevalence might be explained by the current indications for immunotherapy; the tumor types most frequently associated with PNSs (such as small-cell lung cancers, gynecological cancers and thymic tumors) are not generally treated with immune checkpoint inhibitors. Furthermore, PNS are sometime difficult to diagnose and so their prevalence might be underestimated in routine practice [8, 19]. Graus et al. recently stated that the prevalence of neurologic PNS has probably been underestimated as a result of (i) the difficulty of diagnosing these conditions and (ii) the possible underreporting of neurologic irAEs [11]. A recent study of 216 patients with recurrent small-cell lung cancer treated with nivolumab (alone or in combination with ipilimumab) found that four (2%) patients had experienced neurologic irAEs, although the researchers did not state whether or not these events were associated with paraneoplastic features [14]. These data indicate that the neurologic safety of immunotherapy in patients with small lung cancer - a tumor potentially associated with neurologic PNSs - needs to be characterized in more detail.

Our present results showed that PNSs worsened or appeared quite soon after the initiation of immunotherapy; the median time interval was 1.4 months. This finding suggests that patients at risk of a PNS should be closely monitored during the initial immunotherapy cycles. In the present study, we also looked at the causal relationship between anti-PD-1 or anti-PD-L1 immunotherapy and the accentuation or appearance of a PNS. We found that 25% of the patients with a pre-existing PNS and 38% of the patients with a newly diagnosed PNS had obtained an objective tumor response – showing clearly that the PNS was associated with immunotherapy and not with tumor progression.

Neurologic syndromes were the most common and severe PNSs observed in the present study. These neurologic PNSs were of particular concern because of their severity; this might not have been apparent in individual studies or clinical trials but was revealed by our large-scale survey. With regard to the pathogenesis, most studies to date have found that neurologic PNSs have an immune etiology; cross-reactivity occurs when the immune system is misled by the unconventional, ectopic expression of neural proteins on tumor cells [19, 20]. We observed six patients with paraneoplastic encephalitis, and four of the latter were positive for anti-Ma2 autoantibodies. All six patients with encephalitis had severe disease, and three died as a result. The cases of encephalitis were generally difficult to treat, since all patients received various immunomodulatory or immunosuppressive treatments in addition to corticosteroids. Our present data suggest that paraneoplastic encephalitis may be a life-threatening complication of immunotherapy. Importantly, four of the six patients with encephalitis had an objective tumor response at the time when the PNS worsened or appeared, and the other two had stable disease. Hence, the encephalitis was probably triggered by immunotherapy and not by tumor progression. We therefore consider that in clinical practice, (i) all suspected cases of paraneoplastic encephalitis should be investigated extensively (including a screen for anti-neuron antibodies in the serum and cerebrospinal fluid), and (ii) immunotherapy must be discontinued as soon as signs of encephalitis are suspected.

Hypertrophic osteoarthropathy was the second most frequent type of PNS observed in our study; it was generally characterized by digital clubbing, periostitis (often affecting the tibia) and joint pain [21]. In four of the six cases in our study, hypertrophic osteoarthropathy was diagnosed after the initiation of immunotherapy. The condition was mild or moderately severe, and had a limited impact on the patients’ subsequent cancer immunotherapy, which was temporarily discontinued in three cases, and not discontinued in the other three cases. Most of the cases of hypertrophic osteoarthropathy responded well to steroids or nonsteroidal anti-inflammatory drugs, and none of these patients had painful rheumatologic symptoms at the time of analysis. It was not possible to formally establish a causal relationship between immunotherapy and hypertrophic osteoarthropathy, since none of the patients obtained an objective tumor response; hence, the PNS could have been related to tumor progression as well as to immunotherapy.

Dermatomyositis was the third most frequently reported type of PNS; this is a well characterized PNS, particularly when it is associated with anti-TIF1 antibodies [22, 23]. In the present study, we observed five cases of paraneoplastic dermatomyositis. Only one of the five patient with dermatomyositis was seropositive for anti-TIF1 autoantibodies. The paraneoplastic dermatomyositis was severe in all five cases, and had a significant impact on patient care; three of the five patients with dermatomyositis had to permanently discontinue immunotherapy. The cases of dermatomyositis recorded in our study difficult to treat, since four of the five patients required immunomodulatory or immunosuppressive treatments. It is noteworthy that two of the patients with dermatomyositis had an objective antitumor response, indicating that immunotherapy may have had a triggering effect in these individuals.

Overall, one can question the causal relationship between PNSs and immunotherapy. We believe that most of the cases observed in the present study were driven by the tumor, and that immunotherapy merely exacerbated or revealed the clinical symptoms. In cohort 2, the PNSs were detected after the initiation of anti-PD1 or anti-PD-L1 immunotherapy; this prompted us to describe them being incident or newly diagnosed. Given the retrospective nature of the study, however, it is not possible to say whether the PNSs in cohort 2 were present but latent (i.e. clinically asymptomatic) before the initiation of immunotherapy or whether they developed afterwards.

The study’s main limitations were the small number of patients in the final sample and the inherent sources of bias associated with the retrospective, descriptive design. The main bias was selection bias, since patient recruitment was declarative; hence, the most severe cases might have been over-represented in this setting. Consequently, we cannot draw any firm conclusions about the patient distribution. However, given the rarity of PNSs and the often challenging diagnosis, we consider that a declarative study is currently the best way to obtain detailed, useful information. Another limitation was related to data interpretation; it was difficult to distinguishing between a PNS on one hand and a conventional irAE on the other, as these entities can have several common signs and symptoms. To address this issue, we prespecified a list of the most frequent types of PNS (Additional file 1: Table S1) and centrally reviewed all cases of PNS.

## Conclusions

Our present results showed that PNSs tend to be worsened or revealed by anti-PD-1 or anti-PD-L1 immunotherapy. Up to half of patients with a pre-existing PNS might experience a worsening of their symptoms following immunotherapy. Paraneoplastic encephalitis emerged as a potentially life-threatening complication of treatment with immune checkpoint inhibitors. When initiating immunotherapy, physicians should carefully monitor patients with pre-existing PNSs.

## Supplementary information

**Additional file 1: Table S1.** List of different paraneoplastic syndromes predefined in the study by clinical types and categories.

**Additional file 2: Table S2.** Antitumor response rates in patients with a pre-existing PNS (cohort 1).

## Data Availability

Please contact the authors.
