# Supplementary material for: Worsening and newly diagnosed paraneoplastic syndromes following anti-PD-1 or anti-PD-L1 immunotherapies, a descriptive study
Source: J Immunother Cancer. 2019 Dec 3;7:337. doi: 10.1186/s40425-019-0821-8 (PMC6892018; doi:10.1186/s40425-019-0821-8)
Supplement: Supplementary file 1 — Additional file 1: Table S1. List of different paraneoplastic syndromes predefined in the study by clinical types and categories. [file 40425_2019_821_MOESM1_ESM.docx]

**Table S1. List of different paraneoplastic syndromes predefined in the study by clinical types and categories.**

**PNS of renal category** (Paraneoplastic glomerulonephritis documented on renal histology of types):

Membranous nephropathy

Glomerulonephritis with minimal change disease

IgA nephropathy

Glomerulonephritis with focal segmental glomerulosclerosis

Mesangiocapillary glomerulonephritis

Crescentic glomerulonephritis

**PNS of neurological category** (neurological syndromes defined according to the Giometto series; Giometto B, Arch. Neurol. 2010):

Peripheral nervous system

Central nervous system with encephalomyelitis

From the autonomic nervous system (autonomic system disorder, stiff man syndrome…)

Lambert-Eaton syndrome

**PNS of rheumatic category:**

RS3PE syndrome (Remitting seronegative symmetrical synovitis with pitting edema)

Hypertrophic osteoarthropathy (Pierre-Marie et Foix syndrome)

Palmar fasciitis and polyarthritis

Rhizomelic pseudopolyarthritis

**PNS of connective tissue category:**

Paraneoplastic dermatomyositis

Myositis

Vasculitis associated with leukocytoclastic vasculitis type cancer

IgA vasculitis or Henoch-Schönlein purpura

Polyarteritis nodosa

Giant cell arteritis

Paraneoplastic systemic sclerosis

**PNS of cutaneous category:**

Paraneoplastic pemphigus

Acanthosis nigricans defined according to Curth criteria

Ichthyosis acquired

Paraneoplastic Acrokeratosis of Bazex

Erythema gyratum repens

Neutrophilic dermatosis or Sweet's syndrome

**PNS of hematological category:**

Immune Cytopenia Associated with Thymoma or Lymphoid Hemopathy.

**Other paraneoplastic syndrome**

Other, then specify:
