# Supplementary material for: Worsening and newly diagnosed paraneoplastic syndromes following anti-PD-1 or anti-PD-L1 immunotherapies, a descriptive study
Source: J Immunother Cancer. 2019 Dec 3;7:337. doi: 10.1186/s40425-019-0821-8 (PMC6892018; doi:10.1186/s40425-019-0821-8)
Supplement: Supplementary file 2 — Additional file 2: Table S2. Antitumor response rates in patients with a pre-existing PNS (cohort 1). [file 40425_2019_821_MOESM2_ESM.docx]

**Table S2.** Antitumor response rates in patients with a pre-existing PNS (cohort 1).

| Cohort 1: patients with a pre-existing PNS (n = 16 patients) | Exacerbation of a pre-existing PNS during anti-PD1 or PD-L1 immunotherapy? | | p-value# |
| --- | --- | --- | --- |
|  | No (n=8) | Yes (n=8) |  |
| Objective response*, n pts (%)  Complete response  Partial response | 4 (50)  0  4 | 5 (63)  2  3 | p=0.614 |
| No objective response *, n pts (%)  Stable disease  Progressive disease | 4 (50)  2  2 | 3 (37)  1  2 |  |

* The best overall response to anti-PD1 or PD-L1 immunotherapy. Antitumor responses were assessed by the investigators according to the Response Evaluation Criteria in Solid Tumors (version 1.1), as modified for use in clinical trials with immune checkpoint inhibitors.

# Pearson's chi-squared test
